# Comparison of upper body strength gains between men and women after 10 weeks of resistance training

**DOI:** 10.7717/peerj.1627

**Published:** 2016-02-11

**Authors:** Paulo Gentil, James Steele, Maria C. Pereira, Rafael P.M. Castanheira, Antonio Paoli, Martim Bottaro

**Affiliations:** 1 College of Physical Education and Dance, University of Goias, Goiania, Brazil; 2 Centre for Health, Exercise and Sport Science, Southampton Solent University, Southampton, United Kingdom; 3 College of Physical Education, University of Brasilia, Brasilia, Brazil; 4 Department of Biomedical Sciences, University of Padova, Padova, Italy

**Keywords:** Genders, Muscle strength, Peak torque, Elbow flexion, Strength training, Biceps

## Abstract

Resistance training (RT) offers benefits to both men and women. However, the studies about the differences between men and women in response to an RT program are not conclusive and few data are available about upper body strength response. The aim of this study was to compare elbow flexor strength gains in men and women after 10 weeks of RT. Forty-four college-aged men (22.63 ± 2.34 years) and forty-seven college-aged women (21.62 ± 2.96 years) participated in the study. The RT program was performed two days a week for 10 weeks. Before and after the training period, peak torque (PT) of the elbow flexors was measured with an isokinetic dynamometer. PT values were higher in men in comparison to women in pre- and post-tests (*p* < 0.01). Both males and females significantly increased elbow flexor strength (*p* < 0.05); however, strength changes did not differ between genders after 10 weeks of RT program (11.61 and 11.76% for men and women, respectively; *p* > 0.05). Effect sizes were 0.57 and 0.56 for men and women, respectively. In conclusion, the present study suggests that men and women have a similar upper body strength response to RT.

## Introduction

Resistance training (RT) offers benefits to both men and women. Indeed, the health benefits associated with RT include but are not limited to: decreased gastrointestinal transit time; reduced risk of colon cancer (Koffler et al., 1992); increased resting metabolic rate (Campbell et al., 1994; Paoli et al., 2012); improved glucose metabolism (Hurley, 1994); improved blood-lipid profiles (Stone et al., 1982; Hurley et al., 1988); reduced resting blood pressure (Harris & Holly, 1987; Colliander & Tesch, 1988); improved bone mineral density (Menkes et al., 1993); pain and discomfort reduction for those suffering from arthritis (Rall et al., 1996); decreased lower back pain (Deyssig et al., 1993; Weinsier et al., 1995); enhanced flexibility (Westcott, 1995); and, improved maximal aerobic capacity (Steele et al., 2012). In particular, increased strength and muscle mass, the primary outcomes of RT, have been evidenced to reduce the risk of all-cause mortality (Newman et al., 2006; Ruiz et al., 2008; Artero et al., 2011; Srikanthan & Karlamangla, 2014). However, whether there are sex-specific responses to RT is presently unclear. According to Dreyer et al., (2010), in general men have 10 times more circulating testosterone than women. Upper body muscles may have more androgen receptors than lower body muscles (Kadi et al., 2000). Thus, it is possible that this hormonal difference might permit greater development of upper limb muscles in men compared to women with RT.

Janssen et al. (2000) investigated body composition in 268 men and 200 women using magnetic resonance imaging and found upper body muscle mass proportion was greater in men than women (42.9 vs. 39.7%). Similar results were found in resistance-trained subjects by Alway et al. (1989), who compared male and female bodybuilders. They reported that, despite performing the same training volume and performed similar training programs, the muscle cross-sectional area of the biceps brachii was higher in males than in females. Moreover, Kvorning et al. (2006) found that the effects of testosterone on strength gains could be even more pronounced than muscle mass gains. Therefore, it could be hypothesized that sex might influence the strength response more so than the increase in muscle mass in response to RT.

Previous studies reported that men and women performing the same RT regimen did not differ in strength gains, both absolute and relative, for the lower limbs (Ivey et al., 2000; Galvao et al., 2006; Dorgo et al., 2012). Fisher et al. (2014) have recently reported the strength gains of male and female trainees from a private RT facility for both upper and lower body exercises. There were similar absolute strength gains yet females had slightly greater relative strength gains and strength gains relative to body mass. However, females in this study had trained for approximately twice the duration of males. Other studies examining the upper body report conflicting findings with some suggesting greater absolute gains in males (O’Hagan et al., 1995; Abe et al., 2000) and greater relative gains in females. Others reported no differences (Davies et al., 1988; Abe et al., 2000). However, it has been argued by Abe et al. (2000) that many of the earlier studies which conducted sex comparisons for both strength and muscular hypertrophic changes were hampered by low statistical power resulting from small sample sizes (all ≤20 participants).

In considering this, Hubal et al. (2005) conducted a study examining variability in responses and between sex differences to unilateral upper body exercise in a sample of 342 females and 243 males. They reported that, despite there being no significant differences between males and females for absolute strength gains, females had significantly greater relative strength gains compared to males. However, though statistically significant the meaningfulness of these sex differences should be considered. Small differences between data sets can be found to be statistically significant if sample sizes are sufficiently large (Johnson, 1999). The within participant effect sizes calculated using Cohen’s *d* for the changes in both isometric strength and dynamic strength in the study of Hubal et al. (2005) where 14.09 and 28.42 respectively for males and 20.00 and 32.05 respectively for females. Though slightly larger for females, considering the large magnitude of effects for both sexes it is difficult to determine whether the slight differences suggest any meaningful difference.

Studies have confirmed the influence of hormonal differences in muscle mass distribution between men and women, the greater androgen receptors in the upper body musculature, and the supposition that such interactions might influence muscle strength gains in response to RT. Despite this, there is little evidence to suggest sex-specific responses to RT in strength gains of the upper limb. The present research on this topic is potentially limited by both small and large sample sizes, respectively.

Considering the importance of muscle strength to health maintenance and longevity, it is important to further examine sex differences in response to RT to understand the potential efficacy of RT across sexes. As potential differences between sexes could be more evident at the upper body muscles and, considering limitations to investigations comparing upper limb strength gains, the aim of this study was to compare the strength gains of elbow flexors in men and women after 10 weeks of RT.

## Methods

### Participants

Forty-four college-aged men and forty-seven college-aged women were included in the study. Some participants had a few months experience in RT, but none had performed it systematically for more than three months. The volunteers were recruited through folders and advertising banners around the University campus. The criteria for entering the study were being at least 18 years of age, not practicing RT for the past 6 months, and being free of clinical problems that could be aggravated by the study procedures. Participants were instructed to not change their habitual nutritional habits during the study and if any relevant nutritional change was detected (e.g., becoming vegetarian, restricting calories, taking nutritional supplements and/or ergogenic aids), the data for that participant was excluded from the analysis. Initially, 50 volunteers were included in each group. An *a priori* analysis revealed that a sample size of 50 would bring a statistical power of more than 0.9 to detect 10% changes within groups. Data from 9 of them (6 men and 3 women) were excluded from the analysis for failing to meet the inclusion criteria: low attendance (*n* = 6) and performance of additional RT (*n* = 3). All women within the study had regular menstrual cycle’s and testing sessions were performed during the luteal phase of each female participant cycle. All participants were notified of the research procedures, requirements, benefits and risks before providing written informed consent. The Institutional Research Ethics Committee granted approval for the study.

### Peak torque

Unilateral elbow flexion peak torque (PT) was measured using 2 sets of 4 maximal concentric repetitions at 60°/s on a Biodex System 3 isokinetic dynamometer (Biodex Medical Inc., Shirley, NY, USA), with 60 s of rest between sets. Calibration of the dynamometer was performed prior to each testing session in accordance with the manufacturer’s specifications. Participants were seated on a Scott Bench (Gervasport, São Paulo, Brazil) with their elbow aligned with the axis of rotation of the dynamometer’s lever arm. The forearm remained in a supinated position throughout the test. Verbal encouragement was given throughout the test. All tests were administered by the same investigator. Baseline test and retest intraclass correlation coefficient (ICC) for elbow flexors PT was 0.96 (0.93–0.98).

### Resistance training intervention

Men and women performed the same RT protocol, including leg press, knee flexion, chest press and lat pulldowns. Before the beginning of the study, participants performed two weeks of familiarization to find loads to be use during training and to acquaint participants with the exercises and the training program. The RT program lasted for 10 weeks and was performed two days a week, with a minimum of 48 h between sessions. All exercises were performed for 3 sets using a load permitting between 8 and 12 repetitions before achieving momentary muscular failure in order to control for intensity of effort (Steele, 2014). If necessary, loads were adjusted from set to set to maintain the designated range of repetitions. Resistance was increased from session to session by the exercise technician when a subject completed ≥12 repetitions before achieving momentary muscular failure while maintaining proper form. The rest periods between sets were two minutes and the participants were instructed to record training logs for each workout day. Training sessions were closely supervised by experienced trainers, since previous research has demonstrated greater gains in supervised vs. unsupervised training (Gentil & Bottaro, 2010). Furthermore, participants had to attend at least 80% of the training sessions to be included in the analysis (Gentil et al., 2013).

### Statistical analyses

All values are reported as mean ± standard deviation. Normality of distribution for outcome measures was tested using the Kolmogorov–Smirnov test. Data meeting assumptions of normality were subjected to a two-way mixed-factor analysis of variance (ANOVA) to examine the effects of both ‘time,’ ‘sex’ and ‘time × sex’ interaction. Where necessary, multiple comparisons with confidence interval adjustment using Bonferroni procedure were used in the post hoc analysis. Statistical significance was set at *p* ≤ 0.05. Data were analyzed using the *Statistical Package for the Social Sciences* (SPSS) version 17.0 (SPSS Inc., Chicago, IL).

## Results

**Figure 1:**
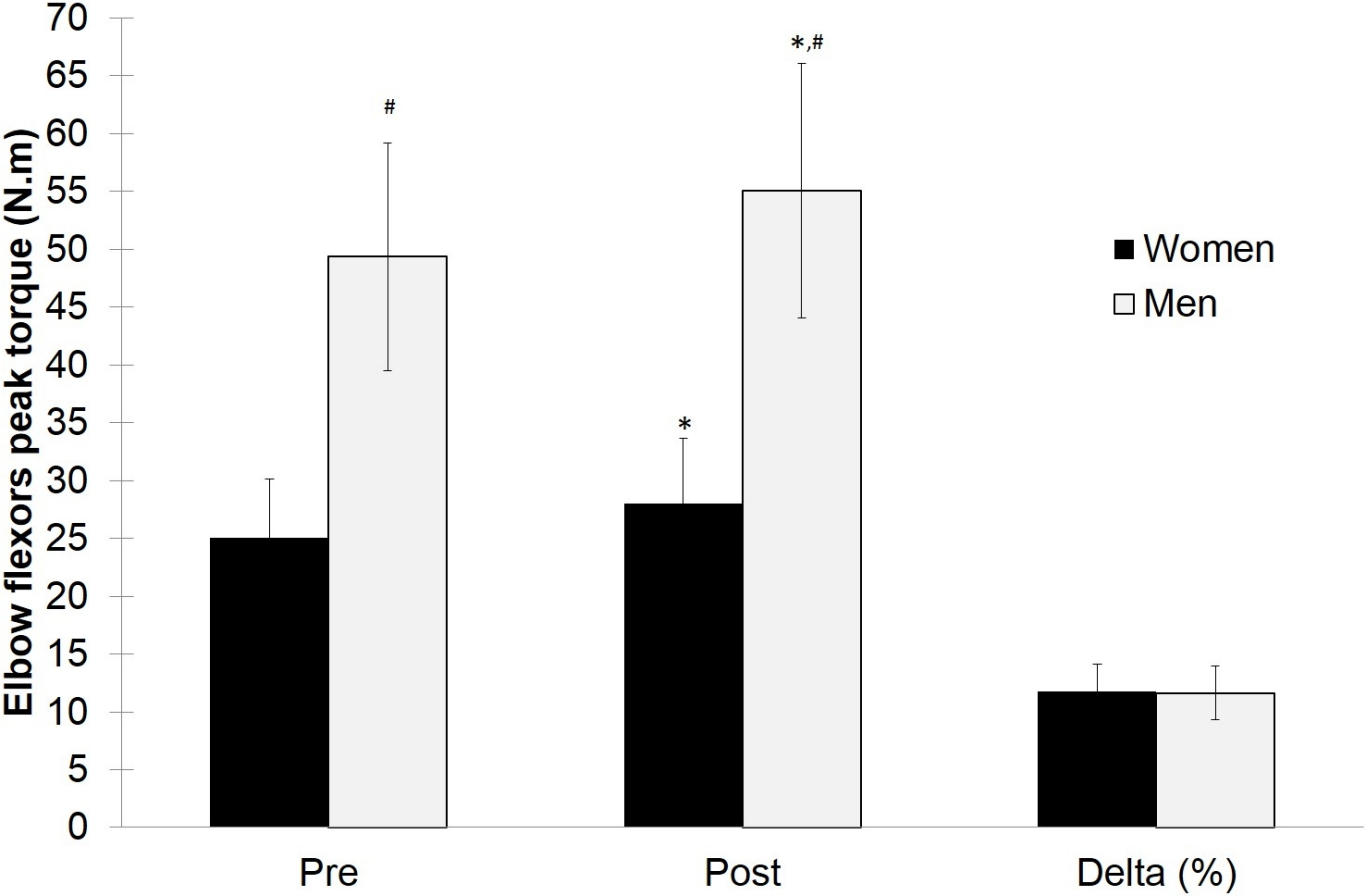
Values and variation in elbow flexor peak torque pre- and post-training period, for men and women. ^∗^ significantly different from pre-training (*p* < 0.05). # significantly different from women (*p* < 0.05).

A total of 91 college students participated in the study; mean values for age, weight and height were 22.01 ± 2.74 years, 66.06 ± 12.16 kg and 168.85 ± 9.25 cm, respectively. Table 1 shows the characteristics of the each group. Peak torque values were greater in men than women in pre- (49.35 ± 10.18 vs. 25.09 ± 4.89) and post-tests (55.08 ± 9.95 vs. 28.04 ± 5.52), as shown in Fig. 1 (*p* < 0.01). Increases in peak torque were significant for both men and women, mean PT 95% CIs [4.36–7.09] and [1.63–4.27], respectively. However, the interaction of sex by time was not significant, suggesting that the alterations in muscle strength were not significant different between sexes (11.61 and 11.76% for men and women, respectively). Cohen’s *d* effect size for PT changes were 0.57 and 0.56 for men and women, respectively. The statistical power to detect PT changes was 99.4% for women and 98.2% for men.

**Table 1:** Subjects characteristics, expressed as means ±standard deviation.

	Men	Women
*N*	44	47
Age (years)	22.63 ± 2.34	21.62 ± 2.96
Weight (kg)	73.06 ± 10.23	58.29 ± 9.00
Height (cm)	174.7 ± 7.10	163.61 ± 7.53

## Discussion

The results of the present study showed no significant differences in elbow flexors strength gains between college-aged men and women after 10 weeks of RT. Our results are in agreement with previous studies of lower body strength gains between sexes in response to the same RT protocol (Ivey et al., 2000; Galvao et al., 2006; Dorgo et al., 2012). Similar results were found for Abe et al. (2000), who evaluated the strength gains of upper and lower body by one-repetition maximum (1RM) tests (bench press and leg extension, respectively). In this study, men and women performed the same RT protocol for 10 weeks and no significant differences (*p* > 0.05) in upper and lower body strength gains were found between sexes.

Although the anabolic and ergogenic effects of testosterone are well known (Kvorning et al., 2006), studies regarding the female sex hormones only became available more recently. Evidence suggests that progesterone may also have an important anabolic effect, which could explain the similar adaptations between sexes (Sakamaki, Yasuda & Abe, 2012; Smith et al., 2014). Sakamaki, Yasuda & Abe (2012) examined elbow extensor muscle volume and isometric strength changes in untrained women during luteal and follicular phases. They found greater isometric strength gains during the luteal phase, when progesterone concentrations were greater. Smith et al. (2014) randomly assigned 24 postmenopausal women to one of four different groups: control (no hormone intervention), testosterone, estradiol or progesterone group treatment. Each hormone regimen was administred to deliver the same amount of hormone concentration of determined phases of the menstrual cycle. In the case of the testosterone group, it was delivered to induce the same hormonal concentration present in women with hyperandrogenemia due to polycystic ovary syndrome. The results showed that progesterone and testosterone induced significant increases of ∼50% in muscle protein fractional synthesis rate, with no effect in the estradiol group. Only the progesterone group had a significant increase of Miogenic Differentiation 1 (MYOD1) mRNA expression an important growth factor regulator related to satellite cells activation. These results might explain why women, even with low circulating testosterone show similar results in relative strength gains when compared with men.

Previous studies showed that female strength, when normalized to muscle mass, is comparable to male values (Hannah et al., 2012) and that the higher male absolute strength is mainly due to higher muscle mass (Ivey et al., 2000). We cannot exclude the possibility that the lack of differences between sexes in the present study may be related to relatively short period of training (10 weeks). One can suggest that the relatively short period of training could be insufficient to produce a significant muscle growth. It is often suggested that the strength increase during the first weeks of training is mainly due to neuromuscular adaptation rather than to muscle mass increase (Moritani & DeVries, 1979; Seynnes, De Boer & Narici, 2007). However, Abe et al. (2000) also reported similar degrees of hypertrophy for males and females occurring as early as ∼6 weeks into an RT intervention with a similar time course of changes for both strength and hypertrophy between sexes.

Further, it could be considered that the sample size used within the present study may have overcome issues relating to both type I and type II statistical errors in earlier studies using both small and large sample sizes (Davies et al., 1988; O’Hagan et al., 1995; Abe et al., 2000; Hubal et al., 2005). Indeed, we also reported similar effect sizes for males and females in our study reinforcing the likelihood that any small differences that may exist between sexes are likely of little practical meaningfulness.

In summary, the present investigation shows that untrained college-aged men and women experience similar elbow flexor strength gains when performing the same RT program for 10 weeks. Despite the physiological and hormonal differences between sexes, women demonstrated the same relative strength gains compared to men in agreement with previous studies. It appears there is presently no evidence of a need to design different RT protocols to men and women. Mostly, one should not expect to find limitations in upper body strength development in women. One limitation of the present study is the lack of body composition analysis. Future studies should attempt to investigate changes in body composition in addition to other potential outcomes from RT in response to the same RT regimen in order to bring more information about sex differences in response to exercise.

## Additional Information and Declarations

### Data Availability

The following information was supplied regarding data availability: Our Ethics Committee does not allow us to share raw data involving human subjects.
